# Determining requirements for patient-centred care: a participatory concept mapping study

**DOI:** 10.1186/s12913-017-2741-y

**Published:** 2017-11-28

**Authors:** Kathryn Ogden, Jennifer Barr, David Greenfield

**Affiliations:** 1 Launceston Clinical School, University of Tasmania, Launceston Clinical School, Locked Bag 1377, Launceston, Tasmania 7250 Australia; 2 Australian Institute of Health Service Management, University of Tasmania, Rozelle Campus, Locked Bag 5052, Alexandria, NSW 2015 Australia

**Keywords:** Concept mapping, Patient-centred care, Health care systems, Implementation, Research

## Abstract

**Background:**

Recognition of a need for patient-centred care is not new, however making patient-centred care a reality remains a challenge to organisations. We need empirical studies to extend current understandings, create new representations of the complexity of patient-centred care, and guide collective action toward patient-centred health care. To achieve these ends, the research aim was to empirically determine what organisational actions are required for patient-centred care to be achieved.

**Methods:**

We used an established participatory concept mapping methodology. Cross-sector stakeholders contributed to the development of statements for patient-centred care requirements, sorting statements into groupings according to similarity, and rating each statement according to importance, feasibility, and achievement. The resultant data were analysed to produce a visual concept map representing participants’ conceptualisation of patient-centred care requirements. Analysis included the development of a similarity matrix, multidimensional scaling, hierarchical cluster analysis, selection of the number of clusters and their labels, identifying overarching domains and quantitative representation of rating data.

**Results:**

The outcome was the development of a conceptual map for the Requirements of Patient-Centred Care Systems (ROPCCS). ROPCCS incorporates 123 statements sorted into 13 clusters. Cluster labels were: shared responsibility for personalised health literacy; patient provider dynamic for care partnership; collaboration; shared power and responsibility; resources for coordination of care; recognition of humanity – skills and attributes; knowing and valuing the patient; relationship building; system review evaluation and new models; commitment to supportive structures and processes; elements to facilitate change; professional identity and capability development; and explicit education and learning. The clusters were grouped into three overarching domains, representing a cross-sectoral approach: humanity and partnership; career spanning education and training; and health systems, policy and management. Rating of statements allowed the generation of go-zone maps for further interrogation of the relative importance, feasibility, and achievement of each patient-centred care requirement and cluster.

**Conclusion:**

The study has empirically determined requirements for patient-centred care through the development of ROPCCS. The unique map emphasises collaborative responsibility of stakeholders to ensure that patient-centred care is comprehensively progressed. ROPCCS allows the complex requirements for patient-centred care to be understood, implemented, evaluated, measured, and shown to be occurring.

**Electronic supplementary material:**

The online version of this article (10.1186/s12913-017-2741-y) contains supplementary material, which is available to authorized users.

## Background

Patient-centredness in health care delivery recognises that patients’ values and preferences must be central in the delivery of care, both at organisational and professional levels [1–4]. Patient-centredness can be considered a necessary attribute for patient-centred care. The notion of patient-centred care dates back to the 1950s [5–7] with multiple efforts to operationalise (see for example: [8–10]) and study [11] it over many decades. However, patient-centred care remains somewhat of an enigma, with “many evangelists but few practitioners” ([12], p. 757). The Institute of Medicine’s ‘Across the Chasm’ report in 2001 called for whole system change, advocating for the need for health care to be patient-centred [13]. There is ongoing debate to the imperative for patient-centred care to be taught and practiced [14–16] and how it can be better achieved [17–21]. Patient-centred care interventions are recognised to positively impact on self-management [22], patient benefits [23], health system quality [24], budget efficiencies [25] and clinical safety [26].

Frameworks, typologies, conceptual maps and domains have been developed for patient-centred care over the past two decades (see for example: [27–31]) in the hope of progressing the application of patient-centred concepts. Common to the existing conceptual frameworks is that the tenets of patient-centredness drawn upon come from academic literature or policy. Sharma and colleagues [11] note that despite the absence of a standard definition, the common guiding components of patient-centred care found within the current literature are: establishing a therapeutic relationship; shared power and responsibility; getting to know the person; empowering the person; trust and respect; and communication.

While there appears to be some agreement of the components of patient-centred care [11, 32] there is a deficit of broad participatory stakeholder engaged research. A consequence is that the task of making patient-centred care a reality, beyond a box ticking exercise, is a challenge for many organisations. There is “a tradition of rhetorical lip service to the centrality of the patient” ([33], p. 1), while rigorous empirical work demonstrating how to *achieve* patient-centred care is lacking. Twelve years on the final Picker Institute report ‘Patient-Centered Care: The Road Ahead’ concludes that patient-centred care remains a slow, elusive achievement [34]. Organisations need practical, clear direction on how to achieve patient-centre care, in short, an answer to the critical question: “what are the organisational *requirements* for patient-centre care?”. The term ‘requirement’ has found its way into the language of systems and business analysis as a “condition or capability needed by a stakeholder to solve a problem or achieve an objective” ([35], p. 1) - something that everyone should know, understand and acknowledge is needed. Our study was derived to meet this need. The research aim was to empirically determine what organisational actions are required for patient-centred care to be achieved.

We approached this task using participatory concept mapping, which allows multiple individual perspectives to be evaluated and integrated [36]. The technique has been identified as one of four methodologies which can drive change within health care systems through their capacity to negotiate complexity and impact the structural and procedural outcomes of transformation [37]. We aimed to build on existing knowledge and practice [38, 39] to develop a conceptual map of what stakeholders require for patient-centred care [40, 41]. The empirically derived map will extend current understandings, create new representations of the complexity of patient-centred care, and be a guide for collective action.

## Methods

### Study design and sampling

We employed a participatory, multi-staged, group concept-mapping approach [42] to address the study’s focus statement: “Patient-centred care requires …”. Concept mapping allows the integration of input from multiple sources with differing perspectives into a conceptual framework, enabling the construction of visual maps with composite thinking of participants or stakeholder groups’ to be represented. The methodology has both face-to-face and online capability, supported by an online platform for the collection, management and analysis of data [43]. The concept mapping methodology used is a recognised method for applied social research. While its origins are in evaluation and planning, it has been used to address health care issues [44, 45]. Group concept mapping is well suited to garnering the views of a broad group of people, has the potential to capture and represent stakeholder groups, is uniquely suited to community-engaged research [36, 46], and is identified as an effective methodology for engaging patients in clinical improvement activities [47].

The study was directed using the methodology described in detail by Kane and Trochim [42], an overview of the steps is as follows. First, brainstorming of ideas, by key stakeholders, using the focus statement resulted in the generation of a list of ideas. Second, these statements were sorted by participants into groups according to similarity, and rated according to three questions pertaining to importance, feasibility and achievement. These questions were chosen to enable organisations to determine priorities (importance), highlight gaps (achievement) for which there may be a greater or lesser ability (feasibility) to address. Third, the resultant data were analysed using quantitative and qualitative techniques to produce a visual concept map representing participants’ conceptualisation of the study topic. Finally, this conceptualisation was presented back to stakeholders for validation.

In order to ensure sampling of the full spectrum of ideas, as opposed to a representative sampling of persons [42], we used a non-random, purposive sampling strategy to identify participants across five stakeholder groups: patients and carers; executives and managers; clinicians and students; educators; and peak body (an Australian term for a group which represents a sector of industry or the community to government) representatives. Participants self-identified their primary stakeholder group, with recognition that many would hold more than one role. Recruitment was achieved via email or through personal request to potential participants who were accessible to researchers through their professional networks and whose knowledge and experience was relevant to the area of enquiry. Only a single request was made to each person. As it is “not necessary that all participants take part in every step of the process” ([42], p. 11), and given our participant group was distributed and broad, potential participants were invited to participate in one or more stages of the project. This study was conducted from August to October 2015 across three of eight Australian states and territories. A detailed description of each step is presented below.

### Generating ideas

Two modes of data collection were used to generate ideas in response to the study focus statement: group brainstorming sessions and contribution of ideas to an online platform [43]. We conducted eight brainstorming groups in two states of Australia. Two groups involved patients and carers only, two groups involved educators (many of whom were clinicians), three groups involved clinicians and students (many of whom were peak group representatives), and one group involved executives and managers. Participants were briefed at the start of the session about the aim of the group – which was to generate ideas relating to the study’s focus statement: ‘Patient-centred care requires …’. We asked participants to complete the focus statement as many times as they liked but not to use the forum to dissect and discuss the ideas in detail. This allowed the brainstorming of ideas regarding the focus statement to remain the central purpose of the groups. The principle of data saturation was used to determine the appropriate sample size for this component of the study. Saturation in naturalistic enquiry entails bringing new participants into the research until a point where the dataset is complete. Knowing when this occurs requires examination of data for replication and redundancy [48]. We determined data saturation through iterative synthesis and comparison of ideas generated at each brainstorming session with those already collected. The online platform was monitored for evidence of new ideas, ideas were synthesised and duplicates removed. This process was conducted collaboratively by two researchers (KO and JB). Once we believed that saturation had been reached we conducted a final multiple stakeholder brainstorming group to confirm no new ideas, refine statements and eliminate statements not relevant to the focus question. Participants who chose to contribute via the online platform responded to the same focus statement as many times as they wished by adding their ideas using a free text response. A set of statements detailing what is required for patient-centred care to be achieved was identified.

### Sorting and rating of statements

Sorting and rating followed the established concept-mapping methodology approach [42, 46]. Participants were invited to sort the statements into groups “in a way that makes sense” to them ([42], p. 72), and provide a relevant name for each group. This activity occurred online using the Concept Systems Global Max^tm^ platform [43]. We set a minimum target of 40 *sorters* with representation from all stakeholder groups, which is more than the recommended number (20–30) to provide reliable results [49]. Pragmatically, we aimed for a total of 40 *raters* with a minimum of five from each stakeholder group, acknowledging that larger number of raters yields higher inter-rater reliability estimates [49]. Participants rated each statement according to three questions:

- How important is this statement to patient-centred care?
  Scale: 1 = not at all important to 5 = extremely important
- How feasible is it for this statement to be achieved?
  Scale: 1 = not at all feasible to 5 = highly feasible
- In your experience, how well is this achieved in Australia?
  Scale: 1 = not at all well to 5 = extremely well.

### Concept-mapping analysis

The analysis employed for this model of concept mapping [42] integrates qualitative and quantitative methods [46, 50] to build a cluster map, label the clusters and to identify overarching domains within the cluster map. Additionally, we used ratings to build go-zones for the map. All quantitative components of the analysis were performed using the Concept System® Global Max^tm^ (build 2013.322.11) software [43].

#### Building the cluster map

A similarity matrix was created, which identifies how often each statement is sorted together. The similarity matrix was then used, through the process of multidimensional scaling [51], to place points representing each statement on a 2-dimensional ‘point map’ to visually represent the sorting data. Statements are placed closer together if sorted together more often. A stress value was calculated to indicate how well the point map represented the raw sorting data [49].

Hierarchical cluster analysis using Ward’s algorithm [52] was used to group statements into clusters representing similar concepts. Cluster analysis is a statistical technique used to develop a higher order understanding of the relationship between statements represented in the point map. The process involves drawing a set of boundaries on the point map to create different numbers of clusters [47]. Bridging values for each statement identified which statements are anchoring -sorted primarily with others close by, and which are bridging - sorted with others across a larger area of the map. It was noted that a large number of statements had high bridging values. Given this, the option of imposing a filter that would require statements to be sorted together by two or more participants was explored. This reduced the tendency for bridging statements, potentially reducing spurious relationships. Further filtering did not reduce bridging statistics therefore the filter was applied.

The process of determining the final cluster number relied on a qualitative analysis by the researchers [46]. Consistent with an interpretive analysis approach [53], we examined the statements in clusters as they were built from maps with five through to 20 clusters. Starting at cluster 5 each cluster was examined to determine the point at which further division of clusters no longer made sense A review of similar studies identified cluster maps with this range [49]. This process of sense making was undertaken by two researchers (KO, JB) independently and then in consultation until agreement was reached on the optimal number of clusters. Using our expertise as clinicians and drivers of education in patient-centred care, the suitability of different cluster solutions to patient-centred care was examined and a decision made for a specific cluster solution based on the content of each cluster and the interrelationships between each item [46]. This cluster representation builds a new collective understanding of individual perspectives [54]. Bridging values were used as a measure of the coherence of a cluster; a set of clusters with low bridging values indicates greater coherence.

#### Determining the names of clusters

Two members of the research team (KO and JB) using information from three sources labelled the clusters collaboratively. The three sources were: the closest fitting labels for each cluster provided by participants; the statement bridging values - to identify those that were most central to the cluster; and the researchers’ understanding of the content of the cluster. By using labels that participants provided to their groupings, we were able to incorporate participant thinking into the process. The team met and discussed this information, working through each cluster to derive an appropriate name as well as considering how the names collectively melded together.

#### Identifying overarching domains

Given the large number of items and clusters representing the complexity of patient-centred care, researchers further reviewed the final cluster map to determine if there was an emergent higher-level organisation of the conceptual model. Through qualitative interpretive analysis of the map, taking into account the proximity of clusters and statements within them, a meaningful interpretation emerged of participants’ lived experiences and contexts regarding patient-centred care [55]. This meaning is presented by grouping the clusters into overarching domains. Given the research context of this study, and a desire to not add participant burden, the cluster solution and domains were determined by researchers. However, following selection of cluster number, naming of clusters and identification of overarching domains, the selection was verified at a series of stakeholder forums where participants had the opportunity to provide input into the final map. The process also served as part of continued stakeholder engagement.

#### Go-zones

Go-zones aid the utilisation of the concept map by visually representing statements within a cluster according to their ratings for importance, feasibility and achievement [42]. A go-zone is a bi-variate graph that plots each statement within a cluster for two of the rated variables. We generated two go-zones for each cluster, one comparing statements’ relative importance and feasibility, and the other comparing importance and achievement. Presenting all rating data and their implications is beyond the scope of this paper. However, a single cluster go-zone is presented to demonstrate the capacity of go-zones to aid utilisation through visual representation of rating data.

## Results

### Participants

There were 91 participants (57 women and 34 men), from across the stakeholder groups, involved in the development of the concept map activities. Forty-six were involved in the validation process (Table 1); 29 of these were also involved in the development phase. Participants came from three of the eight states and territories in Australia. Sixty-one participants identified multiple roles, including a clinical role as part of their responsibilities. Clinician participants included medical, nursing and allied health practitioners, and one clinical ethicist. Those in the patient and carer group all experience, or care for someone who experiences, one or more chronic illness. They had first-hand experience of the acute and community health care sectors. The 68 participants involved in brainstorming activities contributed via online (23), in one of eight sessions (42), and in both ways (3).

**Table 1:** Participant breakdown across the activities

Participant group (designated primary role)	Development of concept map activities						Validation of clusters, labels and overarching domains
	Participants	Brain-storming	Sorting	Importance	Feasibility	How well achieved	
Patients and carers	24	21	11	12	11	10	12
Health care professionals and students	32	25	14	16	12	11	8
Health care educators	18	12	10	12	9	7	10
Management and leadership	10	6	6	5	5	5	7
Peak body representatives	7	4	4	6	2	2	9
Total	91	68	45	51	39	35	46^a^

^a^29 of these participants were also involved in one or more development stage

### The concept map

Participants agreed upon a set of 123 statements to be sorted and rated. The statements were located into a two-dimensional point map with a stress value of 0.248. This value is within the range indicating an acceptable fit between the raw sorting data and the two dimensional configuration [49].

**Fig. 1:**
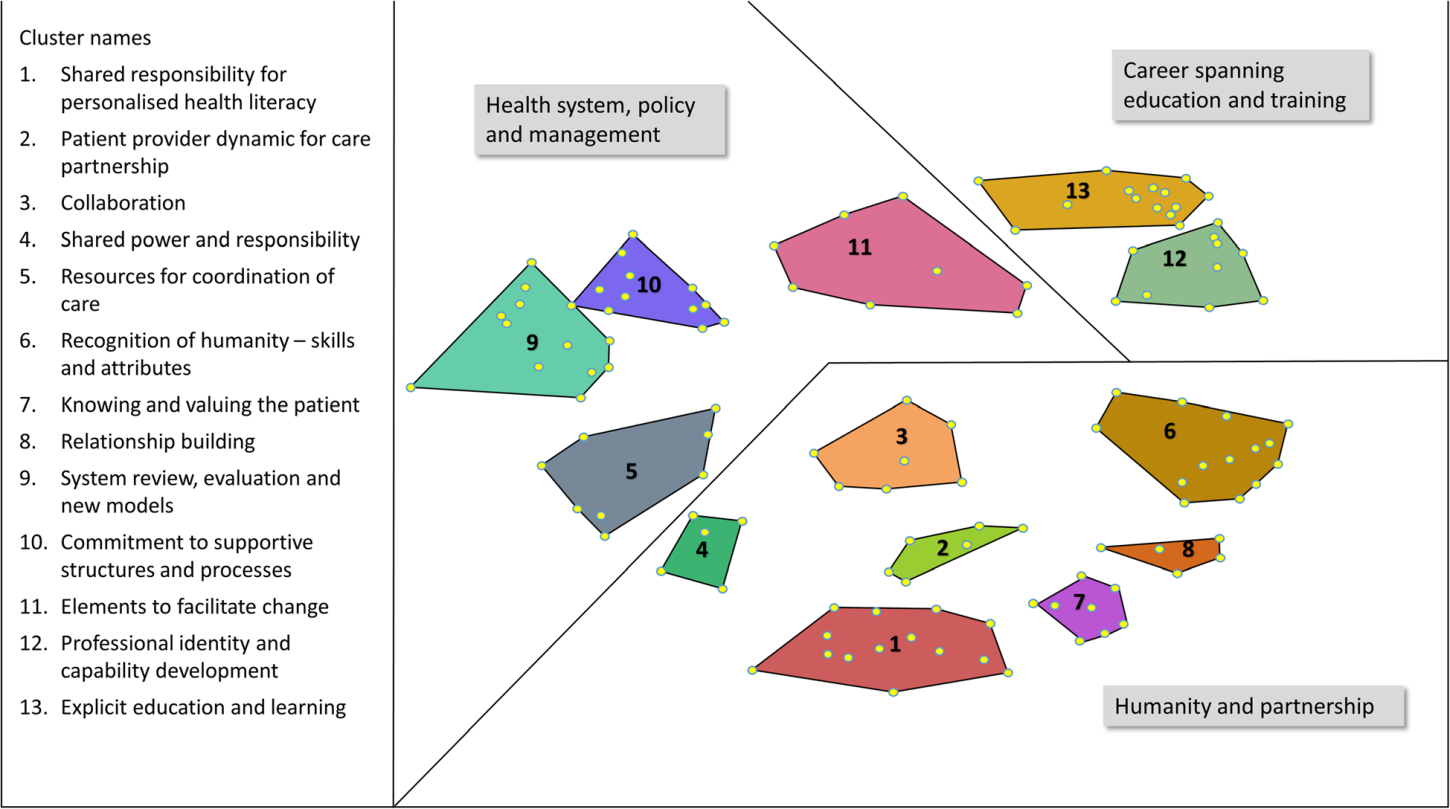
Conceptual map for Requirements of Patient-Centred Care Systems

Hierarchical cluster analysis, in association with interpretive analysis, led to the concept map of 13 clusters for patient-centred care requirements; we name this the ‘Requirements of Patient-Centred Care Systems’ (ROPCCS) conceptual map (Fig. 1). The cluster names shown in the figure legend represent the statements within each cluster, encompassing participants’ understanding of patient-centred care requirements. The map, which represents the requirements for patient-centred care health delivery, was found to display three overarching domains: humanity and partnership; career spanning education and training; and health systems, policy and management.

The elements of the concept map for patient-centred care requirements includes: the 13 clusters and their names; statements within each cluster numbered according to the order they were presented to participants; average ratings for each of the three rating questions; and bridging values [see Additional file 1]. This complete list of statements of the conceptual map is fundamental to the interpretation and meaning given to the overarching map. A summary of the constructs represented by each cluster are presented below (Table 2).

**Table 2:** Description of the elements and constructs within each cluster

Cluster 1: Shared responsibility for personalised health literacy
Encompasses patient and provider responsibilities. The cluster includes: responsibility for personalised care in context; understanding where the patient is at; what level of involvement they want; self-management responsibilities; carer involvement; understanding who the partners are in the care relationship; and, shared decision making by both parties. Fundamental to these requirements is the statement that a level of discussion is necessary to foster knowing, engaging with and collaboration with the patient.
Cluster 2: Patient-provider dynamic for care partnership
Elements in this cluster focus on care requiring a partnership approach. This requires patients and providers acknowledging uncertainty, giving and receiving individualised information and follow-up, explicit goal-setting at each point of care, and adequate time spent negotiating all these aspects.
Cluster 3: Collaboration
Enunciates roles for patient and multidisciplinary providers: trust; respectful understanding; proactive involvement of allied health; undertaking new roles within the care relationship, including a focus on prevention and wellness; and the encouragement of patients' engaging with support networks as part of their overall care plan.
Cluster 4: Shared power and responsibility
Details ideas for improving involvement in care and respecting the patient voice in this process. This includes patient access to health records, patients being health literate, respect between partners, and shared understanding of patient-centred care across sectors. The notion of shared power and responsibility towards and between each party is at the heart of this cluster.
Cluster 5: Resources for coordination of care
Partnership at all levels of the system is ideal, with sharing of patient health records according to patient preferences as a major requirement. Encompasses the need for team approaches, particularly in handover and discharge planning, which includes all members of a patient’s network and an effective advocacy system for those unable to do so for themselves. Transparency of costs and available services are also important requirements.
Cluster 6: Recognition of humanity, skills and attributes
Clinicians and other professional staff require interpersonal skills and attributes that demonstrate attentiveness to individual patients. Necessary for this requirement are excellent communication skills, kindness, listening capabilities that pick up on cues and validate information, being engaged with one another with an awareness of needing to introduce oneself, being empathic and non-judgemental, and having a generalist approach. Understanding end-of-life care and exercising flexible, adaptive capabilities in practice are necessary as well as a willingness to ‘go the extra mile’ in caring for patients.
Cluster 7: Knowing and valuing the patient
Represents elements that may be considered a patient-centred ‘bill of rights’. That is, care according to patient preferences, respecting patient choices and autonomy with consideration of their quality of life, prioritising their management needs and wishes alongside an awareness of all parties’ agendas for care outcomes, encouraging patient participation, building their confidence within the health care environment, and welcoming their lived experiences.
Cluster 8: Relationship building
These elements indicate an essence of curiosity to enable a responsiveness to patients’ values and preferences, including cultural needs, and an understanding of patients’ needs in different clinical circumstances. Having the right relationship between patient and doctor, based on honesty, is required.
Cluster 9: System review, evaluation and new models of care
These elements reflect the complexity of system requirements for patient-centred care to be achieved. This cluster includes: after-hour access; affordability; equity of health care; and adequate resourcing of support services to ensure timely delivery of care. Measurement of patient-centred outcomes and providing feedback of these to stakeholders, and system evaluation to ensure a focus on the patient. Development of new care models are necessary, such as complex care coordinators, longitudinal coordination for care and specific advocacy mechanisms. Health care and community (e.g. schools and workplaces) environments need to be welcoming and safe for patient disclosure.
Cluster 10: Commitment to supportive structures and processes
Consists of organisational mission, structures and processes which are required for patient-centred care. Elements are the removal of barriers for clinicians, organisational philosophy, evidence of compliance to patient-centred care standards in accreditation processes, and the patient voice at executive level of health and educational organisations. Included in this cluster is the requirement for systems that support new models of care.
Cluster 11: Elements to facilitate change
Elements in this cluster refer to the requirement of a best practice approach to facilitate reflection, change and actions for improvements. This means: addressing the culture across stakeholder groups; executive leadership for education and training; establishing ambassadors for change within the health system; developing staff who feel cared for and are empowered for the benefit of patients; training of patients to provide feedback; and providing better evidence for real complexities in patient care. New understandings of power imbalances between patient and health system, and of social determinants of health and their impact on health is required.
Cluster 12: Professional identity and capability development
These requirements span from student education to ongoing learning in the clinical environment. The cluster includes, at the pre-vocational level: teacher role-modelling; specific training for the necessary communication skills; students learning to share evidence and uncertainty with patients; students being positive towards learning from patients; and changing the focus of medical education from ‘doing to’ to ‘doing with.’ In the clinical environment it includes: reflective practice and professional discourse; a focus on avoiding contradictory messages between non-clinical and clinical educational environments; the ability to cope with complexity; maintaining clinical competencies; and understanding that professional development for patient-centred care is required.
Cluster 13: Explicit education and learning
Addressing explicitly the approach to education and learning for patient-centred care is necessary. Elements relate to the explicit teaching of patient-centred care and embedding it within curricula and professional development to build capacity for, and assessment of, humanistic skills. This cluster includes: creating a junior doctor culture which supports doing better for the patient; interdisciplinary learning of roles; involving patients actively in teaching, design, and development of curricula; and longitudinal patient care incorporated in medical education.

### Ratings

The 123 statements, rated by participants on the Likert scale of 1 to 5, received a range of average ratings for importance between 3.04–4.71 (overall mean 3.98); for feasibility between 2.58–4.49 (overall mean 3.61); and for how well achieved between 1.71–3.69 (overall mean 2.56). Ratings for each statement and cluster averages are included in an additional file [see Additional File 1]. For importance, individual statements received a minimum mean value (3.04), which is above the halfway point of three on the scale, indicating that no statement was considered unimportant which further validated our statement list. Feasibility and achievement are likely to be context specific and so not presented in detail. They are used however to demonstrate the capacity of go-zones to the implementation of the conceptual map. Participants perceived clusters located in the ‘humanity and partnership’ domain to be rated more highly than clusters located in ‘health system, policy and management’ domain for importance, feasibility and how well achieved. Clusters in the ‘career spanning education and training elements’ were rated highly for importance and feasibility but lowly for how well achieved. Detailed rating data is beyond the scope of this paper.

### Go-zones

**Fig. 2:**
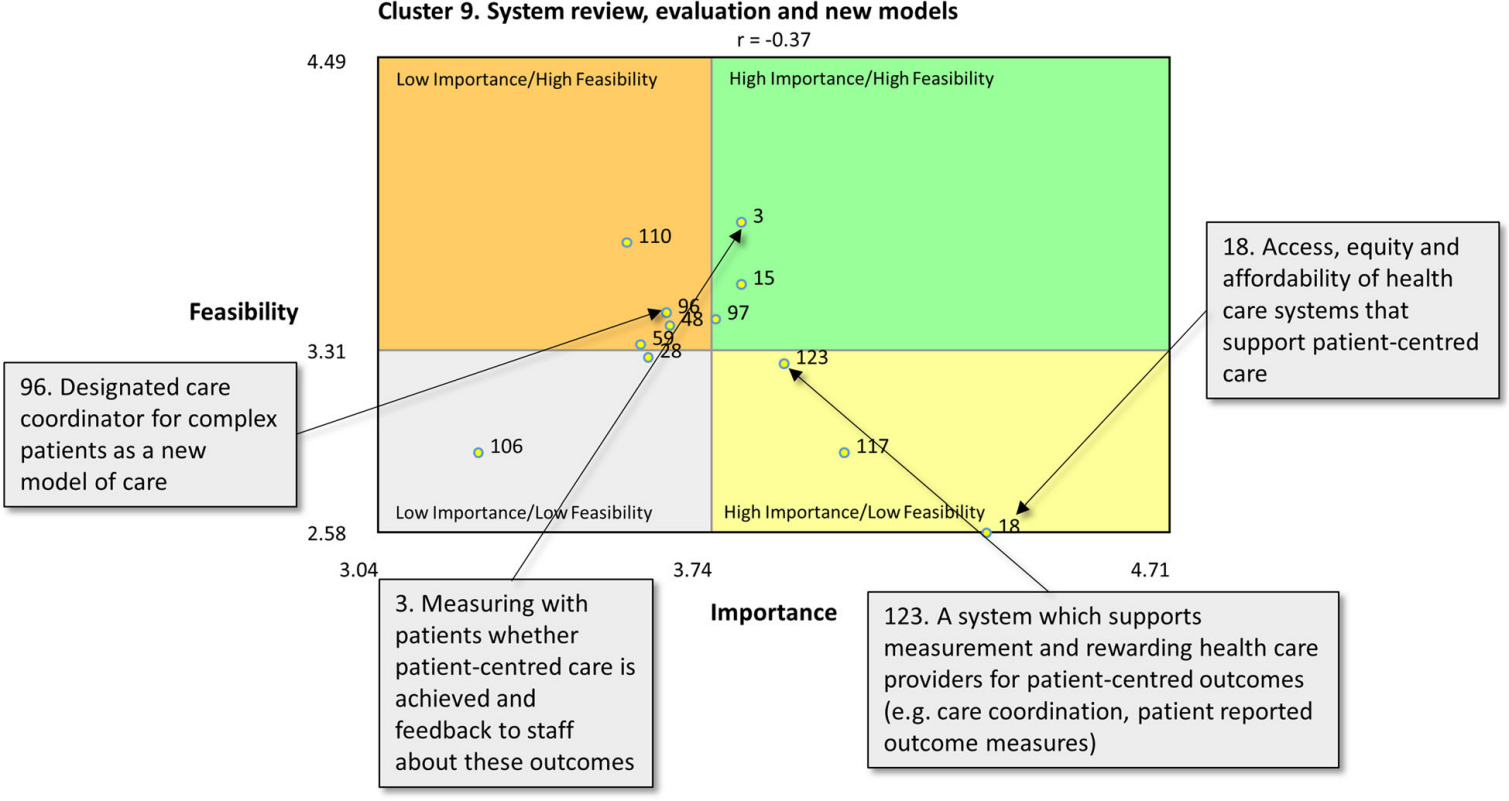
Go-zone for cluster 9: system review, evaluation and new models: Importance vs Feasibility. Scale limits represent lowest (importance = 3.04 and feasibility = 2.71) and highest (importance = 4.71 and feasibility = 4.49) average rating (on a 1–5 scale) for all statements. Cross section value relates to median for that cluster. Statement numbers relate to the order they were presented to participants

**Fig. 3:**
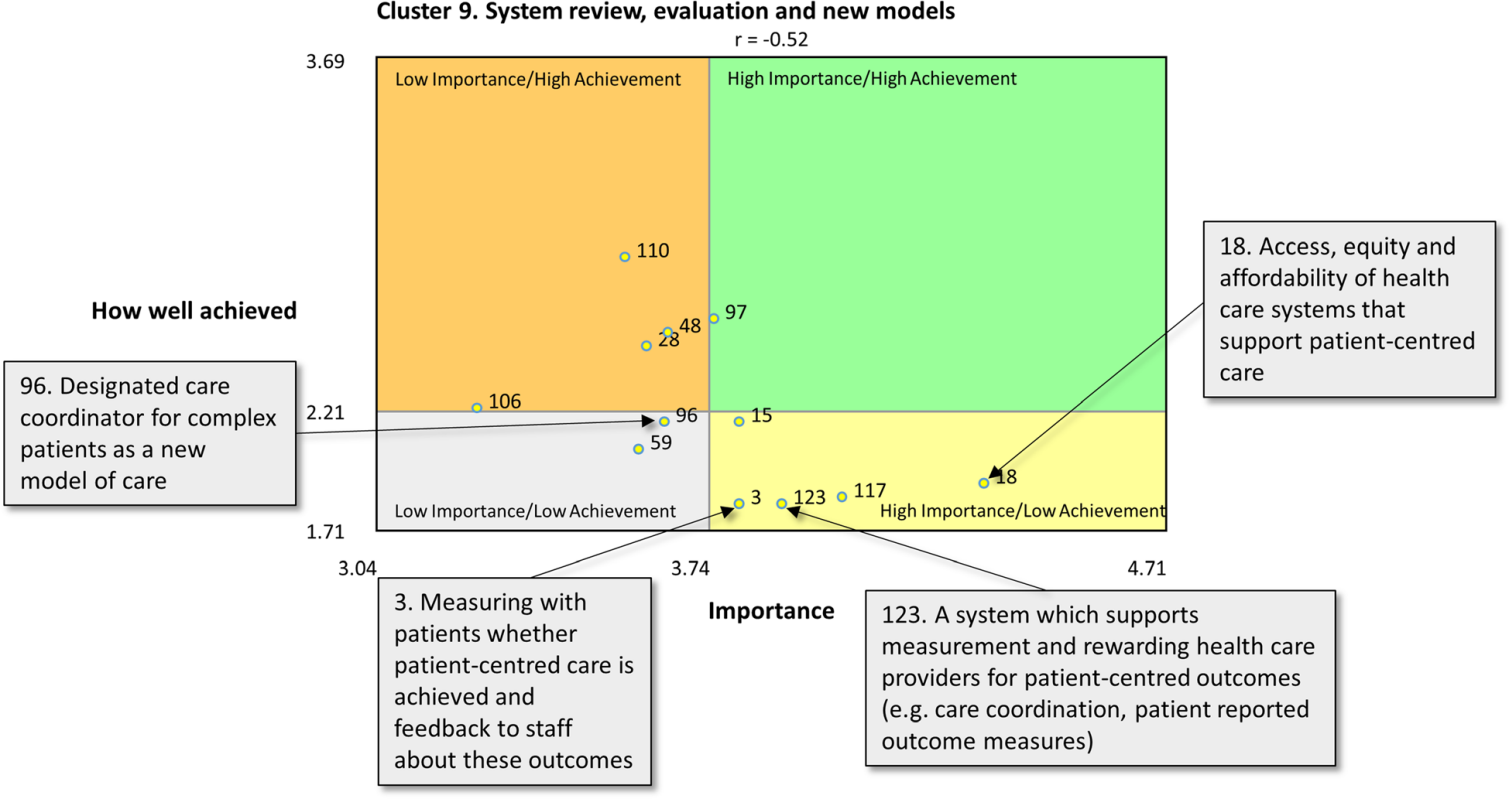
Go-zone for cluster 9: system review, evaluation and new models: Importance vs How Well Achieved. Scale limits represent lowest (importance = 3.04 and how well achieved = 1.71) and highest (importance = 4.71 and how well achieved = 3.69) average rating (on a 1–5 scale) for all statements. Cross section value relates to median for that cluster. Statement numbers relate to the order they were presented to participants

Go-zones were generated for all clusters. The go-zone graphs for cluster 9 ‘systems review evaluation and new models’ are presented for illustration in Fig. 2 (importance vs feasibility) and Fig. 3 (importance vs how well achieved). The figures show how four of the statements within the cluster rated for importance vs feasibility (Fig. 2), and how the same statements rated for importance vs how well achieved (Fig. 3). This visual representation allows statements to be placed in one of four quadrants: high importance and high feasibility or achievement; high importance and low feasibility or achievement; low importance and high feasibility or achievement; and low importance and low feasibility or achievement. The go-zones demonstrated show visually that statement 3, ‘measuring with patients whether patient-centred care is achieved and feedback to staff about these outcomes’, was rated above the mean for importance and feasibility, however well below the mean for how well achieved. Three other statements are also highlighted (Figs. 2 and 3).

## Discussion

Harnessing the experience and views of five stakeholder groups about patient-centred care delivery enabled the creation of the ROPCCS conceptual map. The ROPCCS map presents a unique empirically derived framework of three domains, with 13 clusters and 123 individual statements. At the individual statement level, close examination reveals that many are ‘value’ or ‘position’ statements, which organisations and individual stakeholder representatives need to commit to and work towards constructively. Focusing on what is required of organisations and individuals to action patient-centred care adds an operational micro-perspective enabling application in practice. By making known the ‘requirements’ for patient-centred care, that is, the necessary capabilities or conditions [35], they can be targeted and measured. This will also enable outcomes from actions, activities, or policies to be assessed and improved upon.

The empirically derived ROPCCS conceptual map presents the domains, clusters and statements as *interwoven and at times combined* requirements. The identified domains have added a higher order level of conceptualisation not routinely employed in group concept mapping. We believe that the development of an agreed upon methodology to achieve determination of overarching domains would add value to the method. Illumination of the three overarching domains within the map: humanity and partnership; career spanning education and training; and health systems, policy and management is a key study outcome. The domain arrangement and components of clusters demonstrates the necessity for responsibility, creativity and partnership endeavours between individuals, on differing levels and across settings, for patient-centred care to be realised. This perspective is endorsed by other studies [56] and authoritative commentaries [12, 57] that highlight the multi-dimensional approach necessary if patient-centred care is to be achieved. The clusters of the ROPCCS map reinforce the centrality of productive collaborative relationships, involving constant exchanges between patients, clinicians, managers, students, and educators. When the different actors acknowledge and demonstrate an understanding of one another’s patient-centred care roles and responsibilities, then co-operation and connections become more fruitful. In doing so, the ROPCCS map incorporates and extends the Picker Institute’s eight principles for patient-centred care [29] and highlights Epstein and Street’s [56] belief that implementation of one element of patient-centred care in isolation is too simplistic for effective patient-centred outcomes. ROPCCS differs from other conceptual models [27–31] through its stakeholder participatory research approach, drawing broadly on stakeholder views and experiences and distinguishing itself from literature-based models. This enables the outcomes to be meaningful to all stakeholder groups within health care systems.

Realising patient-centred care will involve the “messy work and complexity of organising change processes in healthcare” ([58], p. 3). Organisational patient-centred cultural change will be productive if every stakeholder position is visible [59], relationship building is deliberately led [60] and every patient-centred care requirement is valued as a shared responsibility [12, 61]. Patient-centredness, transformational leadership and organisational readiness were previously considered to be separate entities, attributes and factors [61] necessary for workplace cultural change. However, Kreindler’s [59] research highlights the propensity for patient-centred care to easily become an ‘inter-group battlefield’ ([59], p. 1139), with conflict between interprofessional, managerial and patient groups on the idea of patient-centred care preventing any collective advancement. ROPCCS allows the potential for stakeholder roles and responsibilities to be visible. The collection of further stakeholder specific rating data will aid understanding for each group within the health system for shared action and responsibility. The need for whole system change, incorporating patient-centred care, is called for [13]; however, now in 2017, a patient-centred care health system remains an elusive achievement [34]. In this way the empirically derived ROPCCS map addresses a critical gap within the literature for a model that encompasses systems and education perspectives to achieve patient-centred care [56]. Systems thinking and complexity science are crucial elements in contemporary approaches to patient-centred care [27, 62–64].

The ROPCCS conceptual map provides the platform for considering patient-centred care strategies from a systems perspective [62, 65, 66]: collaboration across disciplines, sectors (including education) and organisations; ongoing interactive learning; and transformational leadership. Using the ROPCCS map in this way allows creative thinking in four areas: goal setting for education, training and professional development of staff; new strategic directions in care delivery; monitoring and measuring of organisational and care processes, focusing on patient outcomes; and, continuous patient-centred care improvement through analysis of performance and adoption of necessary changes [62, 65, 66]. Future work that will aid this vision is to further examine the causative relationships between individual statements in ROPCCS.

Organisations can be strategically guided by the research outcomes through the ROPCCS map. Implementation of the ROPCCS map will be enabled by gathering organisational specific stakeholders views of how well achieved each statement is, or is not, and their feasibility. Thereby ratings can become context specific and create actionable targets. These ratings can be represented visually using go-zones, which can facilitate constructive discussions between sectors and allow priorities to be determined and targeted through workplace interventions. Hence, organisations can be further guided through measurement of patient-centred care [67, 68], pin-pointing gaps as well as successes, promoting discussion for future planning, and generating ideas for changes to practice [69].

### Limitations

Participant numbers for the brainstorming and sorting components of the study were within the recommended range [49]. The non-random sampling strategy is likely to have resulted in greater participation of advocates for patient-centred care; however we believe that this is appropriate for research of this nature, particularly for the generation of the conceptual map. However, the current participant numbers for ratings were not sufficient to represent reliably the ratings of individual stakeholder sub-groups. The strength of this methodology is that further rating data can be collected from a broader participant group and the findings and implications assessed at a future time.

We acknowledge that this conceptual model for PCC was not generated specifically to meet indigenous Australian PCC requirements. We have consulted with two aboriginal elders and one aboriginal health worker who confirmed that a different set of statements would be necessary for an aboriginal health setting.

## Conclusion

The research has empirically determined what actions are required for patient-centred care to be achieved. The study has developed and presented a patient-centred conceptual map, titled ROPCCS. In doing so we have focussed the current debate on the collaborative responsibility that stakeholders have to ensure that patient-centred care is more comprehensively progressed. Through the derived map the complex requirements for patient-centred care can be understood, implemented, evaluated, measured, and shown to be occurring.

## Additional file

Additional file 1: Requirements of Patient-Centred Care Systems (ROPCCS). The 123 statements of requirements for patient-centred care sorted into clusters with bridging value, and ratings for importance, feasibility and how well achieved for each statement and cluster. (DOCX 68 kb)

## Abbreviations

ROPCCS: Requirements of Patient-Centred Care Systems
