# Supplementary material for: Determining requirements for patient-centred care: a participatory concept mapping study
Source: BMC Health Serv Res. 2017 Nov 28;17:780. doi: 10.1186/s12913-017-2741-y (PMC5704567; doi:10.1186/s12913-017-2741-y)
Supplement: Additional file 1: — Requirements of Patient-Centred Care Systems (ROPCCS). The 123 statements of requirements for patient-centred care sorted into clusters with bridging value, and ratings for importance, feasibility and how well achieved for each statement and cluster. (DOCX 68 kb) [file 12913_2017_2741_MOESM1_ESM.docx]

Requirements of Patient-Centred Care Systems (ROPCCS)

- 123 statements of patient-centred care requirements sorted into 13 clusters.
- Final Stress value is 0.2477after 19 iterations. Filtered by a similarity cut off value=1.
- Each statement and cluster has an allocated bridging value (BV), which relates to whether the statement was sorted primarily with statements close by on the two-dimensional concept map (anchoring statements, low BV), or with statements more distantly located (bridging statements, high BV).
- Average ratings for importance (I), feasibility (F) and how well achieved (HWA) are presented for each statement and cluster. Ratings were made on a likert scale 1-5.
- Statement numbers refer to the order that they were presented to participants.
- Statements are sorted by importance within each cluster.

| **Cluster 1: Shared responsibility for personalised health literacy** | | **I** | **F** | **HWA** | **BV** |
| --- | --- | --- | --- | --- | --- |
| **Cluster average** | | **4.15** | **3.65** | **2.71** | **0.50** |
| 4 | Genuine shared decision making based on individual priorities and preferences and the promotion of self-management, including explanation of the evidence in terms that patients will understand | 4.71 | 3.83 | 2.40 | 0.39 |
| 1 | Individualised care - using evidence judiciously to guide and inform dialogue about what is best for this particular patient and applied in a personalised way | 4.59 | 4.10 | 2.80 | 0.90 |
| 35 | Transparency of medical decision making - patients knowing why decisions are made | 4.39 | 3.87 | 2.94 | 0.39 |
| 38 | A level of discussion which enables knowing, engaging and collaborating with the patient | 4.25 | 3.87 | 2.6 | 0.39 |
| 36 | Patient awareness of all potential implications of treatment/procedures to ensure informed consent | 4.25 | 3.64 | 2.74 | 0.44 |
| 7 | An understanding of patient's social and demographic context and incorporating this knowledge into patient care | 4.24 | 3.60 | 2.8 | 0.57 |
| 88 | Patients understanding who the partners in the care relationship are, and having the care that is being provided explained | 4.17 | 3.64 | 2.51 | 0.49 |
| 102 | The patient having full understanding and ability to participate in the development and implementation of a management plan for them | 4.13 | 3.38 | 2.51 | 0.47 |
| 100 | An understanding that some patients don't wish to make decisions and prefer to defer to family/carer or health care provider | 4.10 | 4.03 | 3.09 | 0.46 |
| 14 | Practitioners assessing patient's health literacy and providing information at an appropriate health literacy level | 4.10 | 3.68 | 2.66 | 0.50 |
| 68 | Family and carer involvement in decision making about care | 4.00 | 3.85 | 3.03 | 0.44 |
| 90 | An exploration of patient capacity for patient involvement | 3.88 | 3.59 | 2.66 | 0.58 |
| 24 | Patient responsibility for an active partnership role in decision making and follow-up | 3.82 | 3.28 | 2.83 | 0.46 |
| 61 | Minimal disruption of the patient's life | 3.48 | 2.77 | 2.34 | 0.54 |

| **Cluster 2. Patient provider dynamic for care partnership** | | **I** | **F** | **HWA** | **BV** |
| --- | --- | --- | --- | --- | --- |
| **Cluster average** | | **4.12** | **3.72** | **2.5** | **0.48** |
| 11 | Adequate time spent consulting with patients to reach a therapeutic alliance and meet their broader health care requirements | 4.35 | 3.15 | 2.17 | 0.50 |
| 77 | Patients to be provided with timely and comprehensive information, in different and appropriate formats, on diagnosis, underlying causes of illness, management and support services | 4.23 | 3.90 | 2.54 | 0.44 |
| 94 | Health care providers and patients acknowledging and managing uncertainty | 4.21 | 3.67 | 2.77 | 0.44 |
| 81 | Follow-up for communication of test results and opportunity for further questions and discussion after initial consultation | 4.06 | 4.18 | 2.94 | 0.46 |
| 30 | Explicit goal setting at every point of care (e.g. acute, primary, palliative) | 3.96 | 3.56 | 2.09 | 0.48 |
| 64 | A method for checking back what a patient takes away from a consultation | 3.92 | 3.87 | 2.49 | 0.56 |
|  | |  |  |  |  |
| **Cluster 3. Collaboration** | | **I** | **F** | **HWA** | **BV** |
| **Cluster average** | | **3.81** | **3.58** | **2.65** | **0.53** |
| 89 | Trust between all partners | 4.33 | 4.08 | 3.03 | 0.48 |
| 26 | Effective and respectful communication between multidisciplinary colleagues | 4.18 | 3.95 | 2.89 | 0.53 |
| 92 | Acceptance that patients have a right to seek a second opinion, in addition to doctors seeking a second opinion when they are unsure | 4.15 | 3.77 | 2.86 | 0.49 |
| 103 | A focus on prevention and wellness | 3.85 | 3.69 | 2.86 | 0.54 |
| 37 | Proactivity of allied health practitioners in patient care | 3.71 | 3.49 | 2.69 | 0.57 |
| 121 | That health care professionals welcome the use by patients of online peer support and sharing of health experiences, and that such sites continue to flourish in a positive and supported manner | 3.43 | 3.36 | 2.40 | 0.59 |
| 16 | Role changes for the patient and provider | 3.04 | 2.73 | 1.83 | 0.53 |
|  |  |  |  |  |  |
| **Cluster 4. Shared power and responsibility** | | **I** | **F** | **HWA** | **BV** |
| **Cluster average** | | **3.98** | **3.55** | **2.42** | **0.76** |
| 6 | Partnership and respect between health-care team members | 4.29 | 3.88 | 2.91 | 0.91 |
| 41 | Mechanisms to give patients a voice at all points of care and in all settings | 4.10 | 3.23 | 1.91 | 0.65 |
| 2 | A shared understanding of patient-centred care (including between patients and their families, clinicians, health care managers, and educators) | 4.06 | 3.73 | 2.43 | 0.69 |
| 67 | Patient's being health literate (seek to develop an understanding of own needs and to understand when to access care) | 3.92 | 3.13 | 2.57 | 0.90 |
| 66 | Patient access to health care records | 3.52 | 3.77 | 2.26 | 0.66 |

| **Cluster 5. Resources for coordination of care** | | **I** | **F** | **HWA** | **BV** |
| --- | --- | --- | --- | --- | --- |
| **Cluster average** | | **3.94** | **3.56** | **2.26** | **0.72** |
| 78 | A team approach towards coordination of care for handover and discharge planning, including patients, family and primary care as key team members and keeping everyone in the loop | 4.27 | 3.82 | 2.34 | 0.61 |
| 122 | Effective patient advocates, especially for those not able to effectively advocate for themselves | 4.04 | 3.54 | 2.49 | 0.84 |
| 5 | Partnership between patient and providers (hospital and community health care facilities) at every level of the system | 4.04 | 2.88 | 1.80 | 0.77 |
| 29 | The sharing of patient records within and between health care sectors according to patient preferences (i.e. deciding who can access records and what information can be shared) | 3.96 | 3.62 | 1.86 | 0.65 |
| 73 | Coordinated ordering of investigations and medications and avoiding unnecessary investigation and treatment | 3.94 | 3.67 | 2.26 | 0.62 |
| 10 | Practitioner and patient awareness of community and societal resources (e.g. allied health, support groups) and being willing to personally interact with them | 3.88 | 3.65 | 2.69 | 0.85 |
| 109 | Patients knowing what health services provides for their particular community groups | 3.71 | 3.67 | 2.49 | 0.83 |
| 40 | Transparency, and consent regarding financial costs, billing and insurance claim systems associated with care | 3.66 | 3.64 | 2.17 | 0.56 |
|  | |  |  |  |  |
| **Cluster 6. Recognition of humanity - skills and attributes** | | **I** | **F** | **HWA** | **BV** |
| **Cluster average** | | **4.21** | **3.82** | **2.98** | **0.55** |
| 22 | Excellent communication skills | 4.59 | 4.05 | 3.03 | 0.62 |
| 42 | Active listening to patients (e.g. being sensitive to responses, picking up on cues, feeding back and validating information) | 4.56 | 4.03 | 2.86 | 0.54 |
| 80 | Empathy towards patients | 4.46 | 4.21 | 3.51 | 0.45 |
| 93 | Understanding the impact of illness on a patient | 4.46 | 3.97 | 2.97 | 0.59 |
| 32 | An engaged health care practitioner | 4.39 | 3.82 | 3.54 | 0.48 |
| 51 | Not being judgemental or making assumptions about patients | 4.36 | 3.64 | 2.74 | 0.53 |
| 74 | A holistic, generalist approach to patient care rather a body part approach | 4.27 | 3.64 | 2.31 | 0.65 |
| 52 | Attitudes that promote and support patient-centred care (e.g. humility, seeing caring for patients as a privilege, kindness) | 4.27 | 3.56 | 2.80 | 0.49 |
| 50 | An understanding of end of life care | 4.20 | 4.18 | 3.26 | 0.63 |
| 9 | Clinicians to be flexible, adaptable and accommodating in their approach to health care planning | 4.18 | 3.58 | 2.43 | 0.52 |
| 107 | Courteous introduction and identification of role | 3.96 | 4.49 | 3.40 | 0.53 |
| 34 | A healthy practitioner | 3.84 | 3.64 | 3.34 | 0.56 |
| 53 | Going the extra mile for patients and following through | 3.82 | 3.36 | 2.77 | 0.47 |
| 105 | A health care team that operates on hope and realistic optimism | 3.50 | 3.38 | 2.77 | 0.60 |
|  | |  |  |  |  |

| **Cluster 7. Knowing and valuing the patient** | | **I** | **F** | **HWA** | **BV** |
| --- | --- | --- | --- | --- | --- |
| **Cluster average** | | **4.16** | **3.68** | **2.67** | **0.45** |
| 19 | A willingness to provide a level of care that fits with the patients overall wishes, and respects patient choices and autonomy, including doing nothing if that's what the patient wants | 4.55 | 3.72 | 2.77 | 0.39 |
| 12 | Consideration of the patient's quality of life | 4.51 | 4.45 | 3.09 | 0.48 |
| 25 | Establishing rapport and a relationship that encourages patient participation, with the ability to express concerns and ask questions | 4.41 | 3.79 | 3.06 | 0.42 |
| 33 | Knowing and prioritising patients' needs and wishes for management | 4.32 | 4.00 | 2.97 | 0.37 |
| 79 | An understanding and acknowledgement of the patient's agenda as well as being mindful of the medical agenda in the patients best interests, ensuring that expectations of service provision align | 4.29 | 3.56 | 2.49 | 0.41 |
| 101 | Welcoming, embracing and valuing patients and their families lived experiences and their expertise in their own management and needs | 4.04 | 3.67 | 2.49 | 0.50 |
| 87 | Building patients' confidence in an alien environment | 3.73 | 3.49 | 2.54 | 0.51 |
| 39 | Clinicians to know the patient's home environment, including by undertaking home visits where possible | 3.41 | 2.74 | 1.94 | 0.51 |
|  | |  |  |  |  |
| **Cluster 8. Relationship building** | | **I** | **F** | **HWA** | **BV** |
| **Cluster average** | | **4.21** | **3.63** | **2.91** | **0.55** |
| 104 | Honesty on the part of patient and health practitioner | 4.48 | 3.85 | 3.4 | 0.57 |
| 54 | A willingness to understand and respond to patient's values, preferences and perspectives | 4.35 | 3.79 | 2.94 | 0.54 |
| 49 | An understanding of different patient needs under different clinical circumstances | 4.20 | 3.92 | 2.97 | 0.51 |
| 17 | A culturally sensitive approach, including spirituality and beliefs, and clinician awareness of own cultural biases | 4.14 | 3.53 | 2.57 | 0.66 |
| 91 | Having the right doctor and the right relationship | 3.88 | 3.08 | 2.66 | 0.46 |
|  | |  |  |  |  |
| **Cluster 9. System review, evaluation and new models** | | **I** | **F** | **HWA** | **BV** |
| **Cluster average** | | **3.74** | **3.31** | **2.21** | **0.51** |
| 18 | Access, equity and affordability of health care systems that support patient-centred care | 4.32 | 2.58 | 1.91 | 0.41 |
| 117 | Adequate resourcing of support services (e.g. drug and alcohol) to ensure timely health care delivery | 4.02 | 2.90 | 1.86 | 0.37 |
| 123 | A system which supports measurement and rewarding health care providers for patient-centred outcomes (e.g. care coordination, patient reported outcome measures) | 3.89 | 3.26 | 1.83 | 0.44 |
| 15 | Periodic review of systems and facilities to ensure patient focus at the individual level | 3.80 | 3.58 | 2.17 | 0.47 |
| 3 | Measuring with patients whether patient-centred care is achieved and feedback to staff about these outcomes | 3.80 | 3.83 | 1.83 | 1.00 |
| 97 | Access to after-hours care | 3.75 | 3.44 | 2.60 | 0.50 |
| 48 | Practical considerations for the physical environment of health care facilities to cater for patients' needs | 3.65 | 3.41 | 2.54 | 0.45 |
| 96 | Designated care coordinator for complex patients as a new model of care | 3.65 | 3.46 | 2.17 | 0.43 |
| 28 | Relational longitudinal coordination of care | 3.61 | 3.28 | 2.49 | 0.61 |
| 59 | Specific mechanism for patient advocacy in acute care systems, the community, to government. Requires an explicit role, independent from health service provider and government, to adequately represent patient views | 3.59 | 3.33 | 2.06 | 0.46 |
| 110 | Health care environments with welcoming, friendly staff and relevant signage as a public declaration of welcome to specific consumer group | 3.56 | 3.74 | 2.86 | 0.53 |
| 106 | Community environments (e.g. schools, workplaces) that allow disclosure of health conditions, according to patient choice, in order to build acceptance, understanding and support networks for the patient | 3.25 | 2.90 | 2.23 | 0.50 |
|  | |  |  |  |  |
| **10. Commitment to supportive structures and processes** | | **I** | **F** | **HWA** | **BV** |
| **Cluster average** | | **3.78** | **3.26** | **2.31** | **0.45** |
| 13 | Removal of barriers for clinicians practising patient-centred care (e.g. financial systems, attitudes) | 4.26 | 2.68 | 1.80 | 0.45 |
| 116 | Minimisation of errors in health care practice | 4.17 | 3.26 | 2.91 | 0.48 |
| 46 | Providers and provider services to recognise the value of patient-centred care through commitment to and communication of a patient-centred mission | 4.06 | 3.77 | 2.66 | 0.47 |
| 21 | A system willing to support and effect change | 3.94 | 2.74 | 2.06 | 0.32 |
| 95 | Accountability at a management/governance level | 3.77 | 3.56 | 2.66 | 0.36 |
| 98 | Shared responsibility between hospitals and their employees for achieving patient-centred care goals | 3.71 | 3.26 | 2.31 | 0.47 |
| 70 | Moving organisational philosophy of health care systems away from the medical model | 3.69 | 2.69 | 1.94 | 0.43 |
| 108 | Processes to be in place in health care services which will identify specific issues that relate to culturally and linguistically diverse groups | 3.69 | 3.51 | 2.66 | 0.44 |
| 60 | The patient voice at executive level of health care and educational organisations | 3.67 | 3.46 | 1.74 | 0.60 |
| 82 | Health care organisation accreditation processes to be based on evidence of compliance to accreditation standards in relation to patient-centred care and partnership in health | 3.58 | 3.67 | 2.63 | 0.45 |
| 84 | Involve consumer groups from the beginning of strategic planning processes so that system design focuses on the service users experience, including design of health care facilities | 3.56 | 3.36 | 2.11 | 0.48 |
| 20 | Different models for conceptualising care | 3.22 | 3.15 | 2.26 | 0.41 |
|  | |  |  |  |  |

| **11. Elements to facilitate change** | | **I** | **F** | **HWA** | **BV** |
| --- | --- | --- | --- | --- | --- |
| **Cluster average** | | **3.67** | **3.44** | **2.36** | **0.70** |
| 47 | Support at an executive level of educational and training institutions | 4.12 | 3.77 | 2.54 | 0.60 |
| 99 | Staff who feel cared for, know the joy of coming to work and empowered to do things for patients | 3.85 | 3.15 | 2.23 | 0.62 |
| 119 | Better evidence that relates to real world patients and their complexities | 3.74 | 3.44 | 2.43 | 0.86 |
| 8 | An understanding of power imbalance between patient groups and the health care system | 3.65 | 3.58 | 2.06 | 0.69 |
| 62 | A comprehensive understanding of the social determinants of health and how they impact health | 3.63 | 3.51 | 2.83 | 0.66 |
| 115 | Training consumers about how to provide feedback and raise issues | 3.58 | 3.59 | 2.26 | 0.81 |
| 65 | Ambassadors for change in health care systems | 3.47 | 3.26 | 2.17 | 0.63 |
| 83 | Adequate orientation to, and general support of, international health care providers in the local environment | 3.33 | 3.18 | 2.40 | 0.70 |
|  | |  |  |  |  |
| **12. Professional identity and capability development** | | **I** | **F** | **HWA** | **BV** |
| **Cluster average** | | **4.12** | **3.87** | **2.79** | **0.32** |
| 43 | Teaching and leading by example through role modelling to students and junior staff | 4.38 | 4.10 | 2.83 | 0.21 |
| 23 | Specific teaching and training for the required communication skills | 4.35 | 4.26 | 2.86 | 0.32 |
| 120 | Teaching students to share evidence and uncertainty with patients in a manner which is able to be understood by patients by avoiding jargon and using patient friendly research terms that are meaningful to patients (e.g. absolute risk, number needed to treat) | 4.28 | 4.05 | 2.83 | 0.20 |
| 31 | Maintenance of clinician's professional competencies and being up to date | 4.20 | 4.18 | 3.69 | 0.44 |
| 86 | An understanding that patient-centred care is not an innate capability and requires professional development | 4.06 | 4.18 | 2.54 | 0.18 |
| 112 | The avoidance of contradictory messages between the non-clinical and clinical learning environment | 4.06 | 3.03 | 2.46 | 0.50 |
| 114 | Positive attitudes from students towards patient-centred learning (learning with and from patients) | 4.06 | 3.90 | 3.11 | 0.08 |
| 76 | A change of the focus of medical education from 'doing to', to 'doing with' | 4.00 | 3.87 | 2.46 | 0.30 |
| 69 | The capability of health care professionals to cope with complexity of health and health care systems | 3.98 | 3.44 | 2.49 | 0.51 |
| 27 | Reflective practice of health care providers and students, including opportunities for professional discourse | 3.80 | 3.69 | 2.66 | 0.48 |
|  | |  |  |  |  |

| **13. Explicit education and learning** | | **I** | **F** | **HWA** | **BV** |
| --- | --- | --- | --- | --- | --- |
| **Cluster average** | | **3.93** | **3.81** | **2.49** | **0.17** |
| 57 | Commitment of university and training colleges to ensure patient-centred education is embedded in curricula | 4.41 | 4.28 | 2.57 | 0.01 |
| 45 | Educators to recognise the value of patient-centred care | 4.38 | 4.31 | 3.31 | 0.11 |
| 55 | Professional development to build the capacity for humanistic skills required for patient-centred care in health professionals from graduation through careers | 4.16 | 3.77 | 2.40 | 0.10 |
| 63 | A culture for junior doctors that supports a desire to do better according to patient's needs and concerns and avoids fear of negative ramification | 4.14 | 3.56 | 2.23 | 0.49 |
| 44 | Explicit teaching of patient-centred care with consideration of appropriate staging of skill development and repeated opportunities for practice | 4.14 | 4.15 | 2.71 | 0.11 |
| 111 | A safe and comfortable learning environment for both student and patient to engage in patient-centred learning | 3.96 | 4.00 | 2.97 | 0.16 |
| 75 | Medical education that incorporates longitudinal patient care | 3.96 | 4.05 | 2.66 | 0.00 |
| 58 | Recognising and including competencies related to patient-centred care in vocational training selection process | 3.94 | 3.87 | 2.34 | 0.00 |
| 56 | Emphasis on observation and work-place assessment of humanistic skills in trainees | 3.84 | 3.72 | 2.34 | 0.03 |
| 71 | The involvement of patients in medical education, including active teaching roles | 3.84 | 3.87 | 2.57 | 0.20 |
| 113 | Making acute care settings explicitly suitable for patient-centred teaching | 3.73 | 3.21 | 2.20 | 0.53 |
| 118 | Opportunities for interdisciplinary students to learn and work together and to develop understanding of each other's roles | 3.71 | 3.51 | 2.34 | 0.32 |
| 85 | Early capacity training in cultural awareness | 3.60 | 3.90 | 2.51 | 0.15 |
| 72 | The involvement of patients in design and development of curriculum for education and training | 3.24 | 3.15 | 1.71 | 0.20 |
